# Supplementary material for: Effect of Different Probiotic Fermentations on the Quality of Plant-Based Hempseed Fermented Milk
Source: Foods. 2024 Dec 17;13(24):4076. doi: 10.3390/foods13244076 (PMC11675659; doi:10.3390/foods13244076)
Supplement: Supplementary file 1 [file foods-13-04076-s001.zip › foods-3320290-supplementary.pdf]

Effect of different probiotic fermentations on the quality of hemp seed plant-based yogurt

Yingjun Zhou[1], Yifan Xu[1#], Shuai Song[2], Sha Zhan[1], Xiaochun Li[1], Haixuan Wang[2], Zuohua Zhu[1], Li Yan[1], Yuande Peng[1], Chunliang Xie[1]*

[1] Institute of Bast Fiber Crops, Chinese Academy of Agricultural Sciences, Changsha 410205, China

[2] Jiangsu junyao Life Technology Development Co., Ltd., Jiangsu 224100, China

* Correspondence.

E-mail: xiechunliang@caas.cn

Table S1. Results of volatile flavor components of samples.

Compound	Relative Amount				Odor Description
	HMD	HMP	HMR	HMC	
**Aldehydes**					
Propanal	5.19±0.64%	4.67±0.12%	7.75±0.09%	4.34±0.07%	pungent, green grassy
Acetaldehyde	3.84±0.07%	4.28±0.21%	5.00±0.23%	2.93±0.05%	green, slight fruity
Hexanal	1.85±0.64%	3.51±0.07%	7.01±0.2%	3.1±0.17%	fresh, green, fat, fruity
(E)-2-Pentenal	1.06±0.17%	1.76±0.02%	1.22±0.08%	2.08±0.02%	potato, peas
3-Methylbutanal	0. 50±0.06%	0.22±0.01%	1.00±0.06%	0. 42±0.01%	chocolate, fat
(E)-2-Heptenal	0.56±0.06%	0.94±0.02%	0.97±0.06%	0.48±0.03%	spicy, green vegetables, fresh, fatty
(E)-2-Hexenal	0.54±0.09%	0.86±0.01%	0.82±0.03%	0.48±0.01%	green, banana, fat
Pentanal	0.11±0.03%	0.22±0.01%	0.90±0.03%	0.11±0.01%	green grassy, faint banana, pungent
2-Methylbutanal	0.40±0.04%	0.23±0.03%	0.61±0.06%	0.23±0.01%	almond, cocoa, malt
Butanal	0.33±0.02%	0.29±0.02%	0.63±0.01%	0.20±0.01%	pungent, fruity, green leaf
(E)-2-Octenal	0.22±0.05%	0.50±0.01%	0.21±0.00%	0.32±0.01%	fresh cucumber, fatty, green herbal, banana, green leaf
3-Methyl-2-butenal	0.35±0.00%	0.23±0.01%	0.24±0.01%	0.25±0.01%	fruity
Benzaldehyde	0.20±0.02%	0.15±0.01%	0.21±0.02%	0.20±0.01%	bitter almond, cherry, nutty
Heptanal	0.21±0.03%	0.15±0.01%	0.26±0.01%	0.20±0.01%	fresh, aldehyde, fatty, green herbs, wine, fruity
Nonanal	0.12±0.01%	0.10±0.02%	0.12±0.02%	0.09±0.01%	rose, citrus, strong oily
(E,E)-2,4-Heptadienal	0.13±0.02%	0.20±0.02%	0.17±0.01%	0.13±0.01%	fatty, oily, aldehyde, vegetable, cinnamon
2-Propenal	0.09±0.00%	0.16±0.01%	0.12±0.01%	0.11±0.00%	strong pungent
2-Methylpropanal	0.11±0.01%	0.03±0.00%	0.10±0.01%	0.06±0.00%	banana, melon , slightly nutty
2-Methyl- 2-pentenal	0.05±0.00%	0.06±0.00%	0.07±0.01%	0.10±%0.01	aldehydes, soil, garlic, ripe cherries, fruity
**alcohol**					
Ethanol	15.74±0.32%	11.1±0.11%	11.23±0.16%	11.69±0.2%	aromaticity
1-Hexanol	3.62±0.28%	5.93±0.06%	2.59±0.09%	3.31±0.09%	fresh, fruity, wine, sweet, green
1-Propanol	3.32±0.1%	1.44±0.02%	1.58±0.02%	1.84±0.02%	alcohol, pungent
1-Penten-3-ol	2.16±0.12%	2.44±0.05%	1.99±0.04%	2.97±0.06%	ethereal, green, tropical fruity
1-Pentanol	1.23±0.08%	1.72±0.01%	1.49±0.03%	2.24±0.02%	balsamic
3-Methyl-1-butanol	0.97±0.03%	0.94±0.01%	0.82±0.01%	0.95±0.02%	whiskey, banana, fruity
1-Butanol	0.82±0.05%	0.83±0.03%	0.81±0.02%	1.02±0.04%	wine
2-Propanol	0.47±0.03%	0.60±0.01%	0.44±0.02%	0.41±0.02%	alcohol, spicy
2-Methyl-1-propanol	0.41±0.02%	0.63±0.01%	0.28±0.01%	0.37±0.01%	fresh, alcoholic, leather
1-Octen-3-ol	0.17±0.02%	0.29±0.00%	0.23±0.02%	0.29±0.00%	mushroom, lavender, rose, hay
1-Heptanol	0.12±0.01%	0.15±0.01%	0.14±0.01%	0.27±0.01%	grape, fruity, wine, violet, peony
3-Methyl-3-buten-1-ol	0.14±0.00%	0.27±0.01%	0.12±0.01%	0.16±0.00%	sweet, fruity
2-Heptanol	0.13±0.01%	0.17±0.01%	0.14±0.01%	0.10±0.00%	mushroom、melon
2-Butanol	0.14±0.00%	0.12±0.00%	0.10±0.01%	0.16±0.00%	fruity
2-Pentanol	0.06±0.01%	0.07±0.00%	0.07±0.01%	0.13±0.01%	Fusel Oil, Green
**ketone**					
2-Propanone	8.43±0.47%	8.81±0.14%	8.86±0.13%	8.51±0.14%	fresh, apple, pear
3-Hydroxy-2-butanone	4.87±0.2%	6.49±0.13%	3.87±0.1%	4.4±0.19%	fatty, oily, aldehyde, vegetable, cinnamon
2-Pentanone-D	3.70±0.39%	4.07±0.05%	3.71±0.07%	5.16±0.04%	acetone, fresh, sweet fruity, wine
2-Butanone	3.27±0.18%	3.26±0.05%	4.96±0.08%	4.62±0.17%	fruity , camphor
2,3-Butanedione	1.15±0.09%	1.26±0.02%	1.45±0.07%	0.71±0.02%	butter, popcorn, sweet taste, sour rice
2-Heptanone-M	1.22±0.06%	0.78±0.12%	1.64±0.02%	0.18±0.00%	pear, banana, fruity, slight medicinal fragrance
1-Penten-3-one	0.36±0.04%	0.79±0.02%	0.24±0.01%	0.29±0.01%	strong pungent odors
2-Hexanone	0.35±0.01%	0.32±0.01%	0.33±0.01%	0.25±0.00%	fruity, fungal, meaty, buttery
2,3-Pentanedione	0.26±0.02%	0.25±0.03%	0.28±0.01%	0.28±0.02%	sweet, cream, caramel, nuts, cheese
4-Methyl 2-pentanone	0.25±0.02%	0.25±0.01%	0.23±0.00%	0.24±0.02%	ketone
2-Nonanone	0.07±0.01%	0.07±0.01%	0.08±0.01%	0. 16±0.01%	fresh, sweet, green, herb
**alkene**					
Myrcene	0.16±0.02%	0.25±0.00%	0.15±0.01%	0.35±0.01%	must, spice, balsamic

3-Carene	0.12±0.01%	0.15±0.02%	0.10±0.01%	0.13±0.00%	citrus, lemon, woody
beta-Pinene	0.05±0.00%	0.07±0.00%	0.04±0.00%	0.26±0.01%	resin, green
**ester**					
Ethyl acetate	9.24±0.89%	6.88±0.5%	8.55±0.12%	9.34±1.2%	fresh, fruity, sweet, grassy
Ethyl butanoate	0.30±0.03%	0.31±0.01%	0.38±0.01%	0.30±0.01%	pineapple, fruity, ester, whiskey
cis-3-Hexenyl acetate	0.08±0.01%	0.05±0.01%	0.06±0.01%	0.06±0.01%	fresh green grassyy, sweet, fruity, banana
**carboxylic acid**					
Acetic acid	16.29±1.05%	17.43±0.07%	12.32±0.36%	19.22±0.57%	spicy
Propanoic acid	0.47±0.03%	0.51±0.02%	0.53±0.04%	0.54±0.02%	yogurt, vinegar
**sulfide**					
Dimethyl sulfide	2.05±0.09%	0.80±0.05%	0.79±0.15%	1.09±0.05%	cabbage, sulfur, gasoline
Dimethyl disulfide	0.02±0.00%	0.04±0.00%	0.05±0.01%	0.05±0.01%	sulfurous , cabbage, onion
**furan**					
2-Pentylfuran	0.06±0.01%	0.15±0.01%	0.06±0.01%	0.18±0.01%	bean, fruity, earthy, green, vegetable
**Others**					
Ethylbenzene	0.11±0.00%	0.12±0.01%	0.12±0.03%	0.24±0.02%	Aromatic odor
2-Ethylpyrazine	0.11±0.01%	0.11±0.00%	0.09±0.01%	0.09±0.01%	nutty, mouldy, woody, potato, earthy, roast, meat, fishy, cocoa
1,8-Cineole	0.08±0.01%	0.1±0.01%	0.09±0.00%	0.06±0.00%	camphor, refreshing herbal
